# Supplementary material for: Large-scale HLA immunopeptidome and interactome profiling in microglia
Source: bioRxiv. 2025 Apr 26:2025.04.23.650327. Preprint. [Version 1] doi: 10.1101/2025.04.23.650327 (PMC12190351; doi:10.1101/2025.04.23.650327)

Supplementary Figure 1

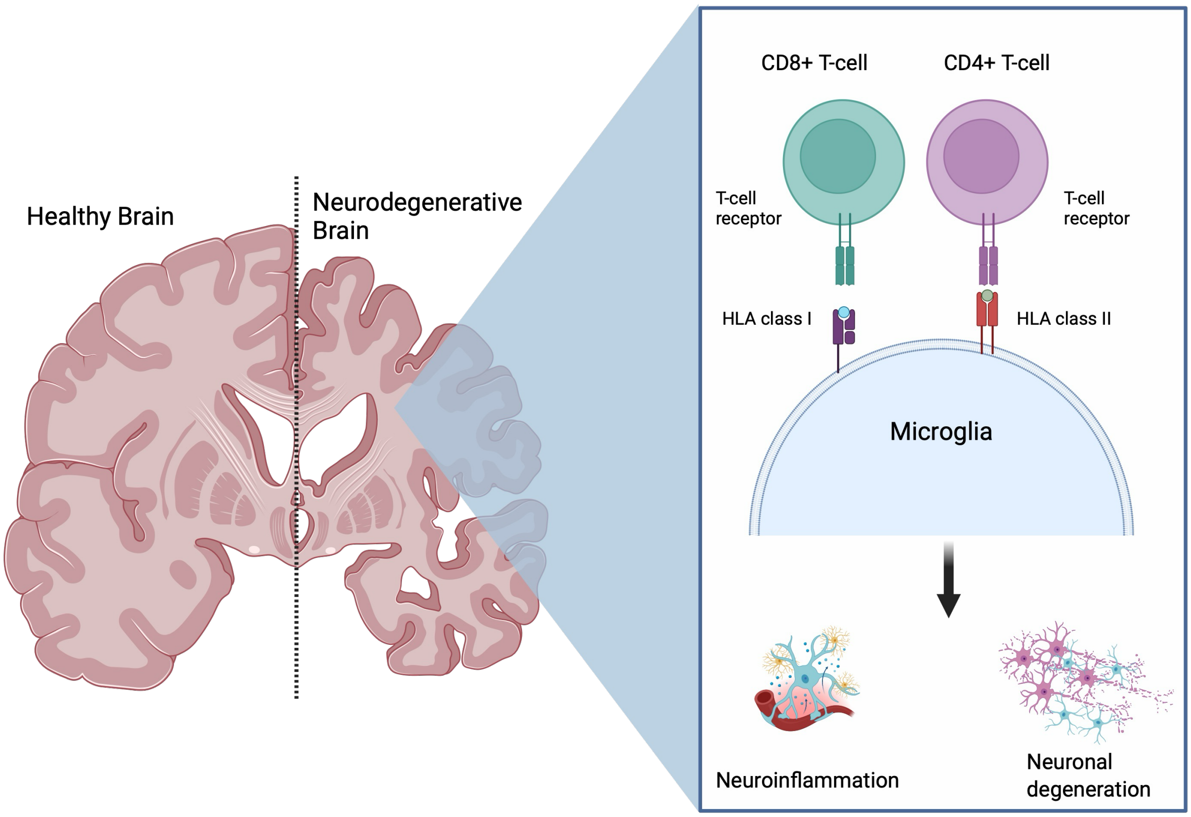

Supplementary Figure 2

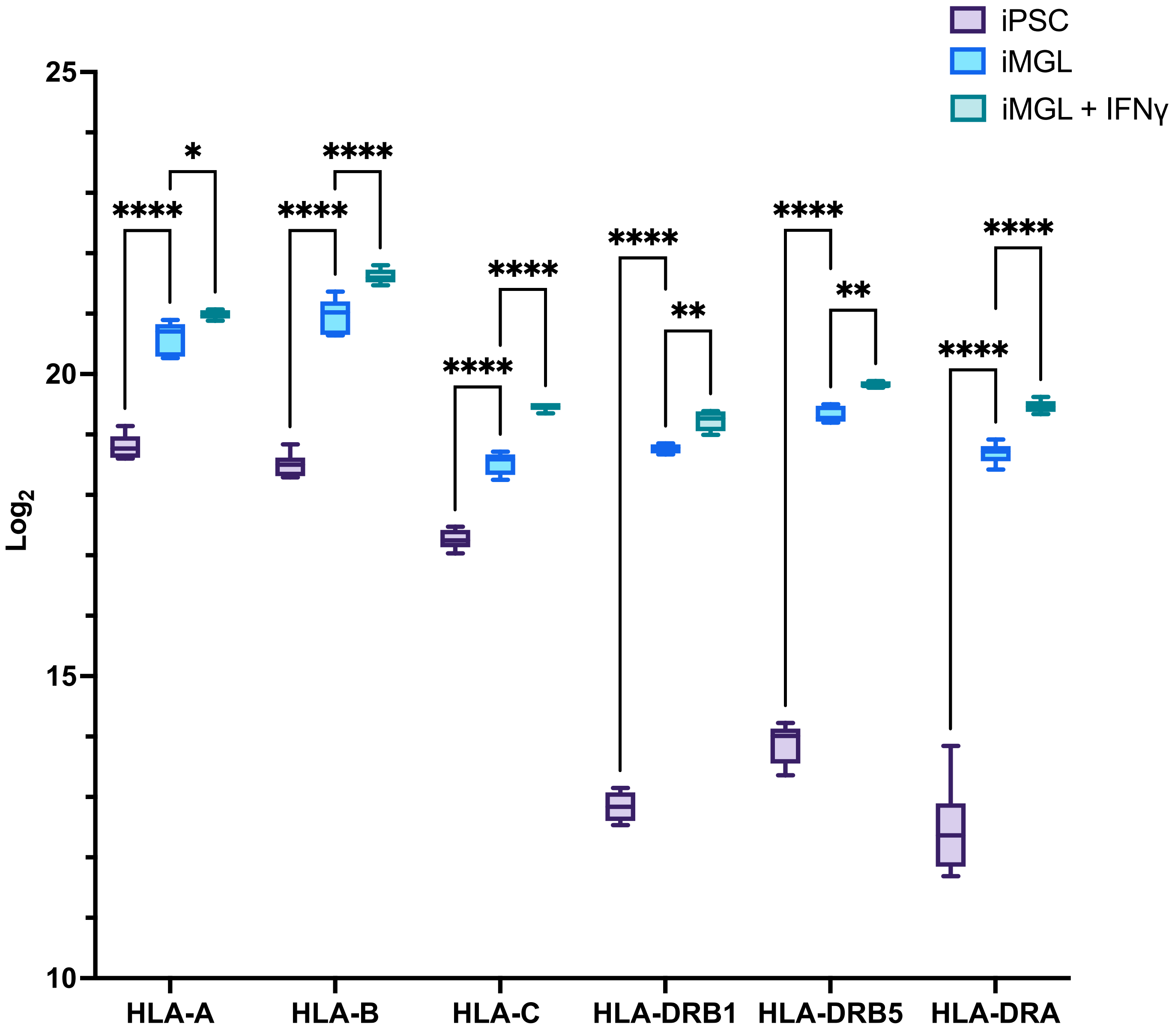

Supplementary Figure 3

A)

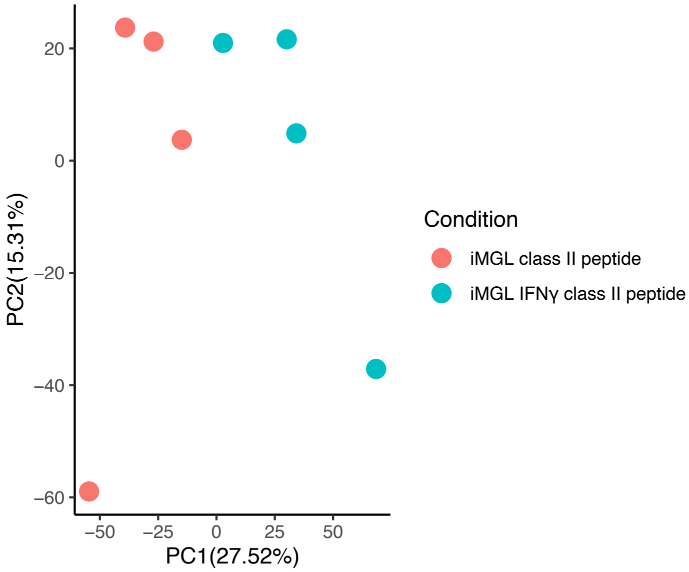

B)

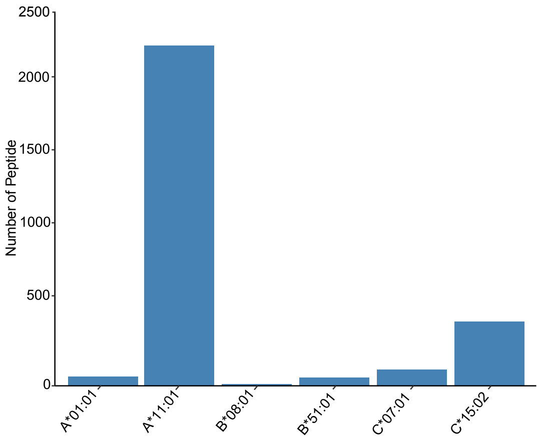

C)

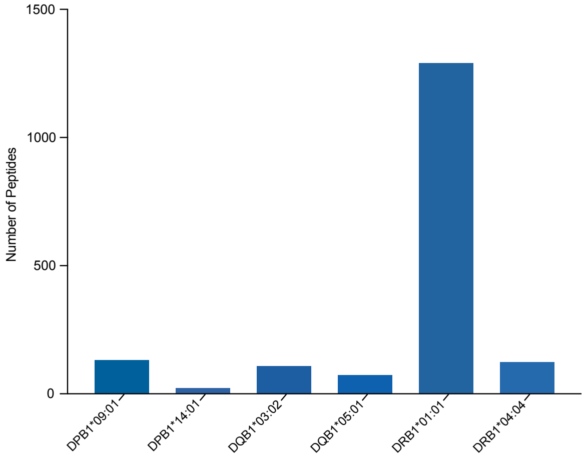

Supplementary Figure 4

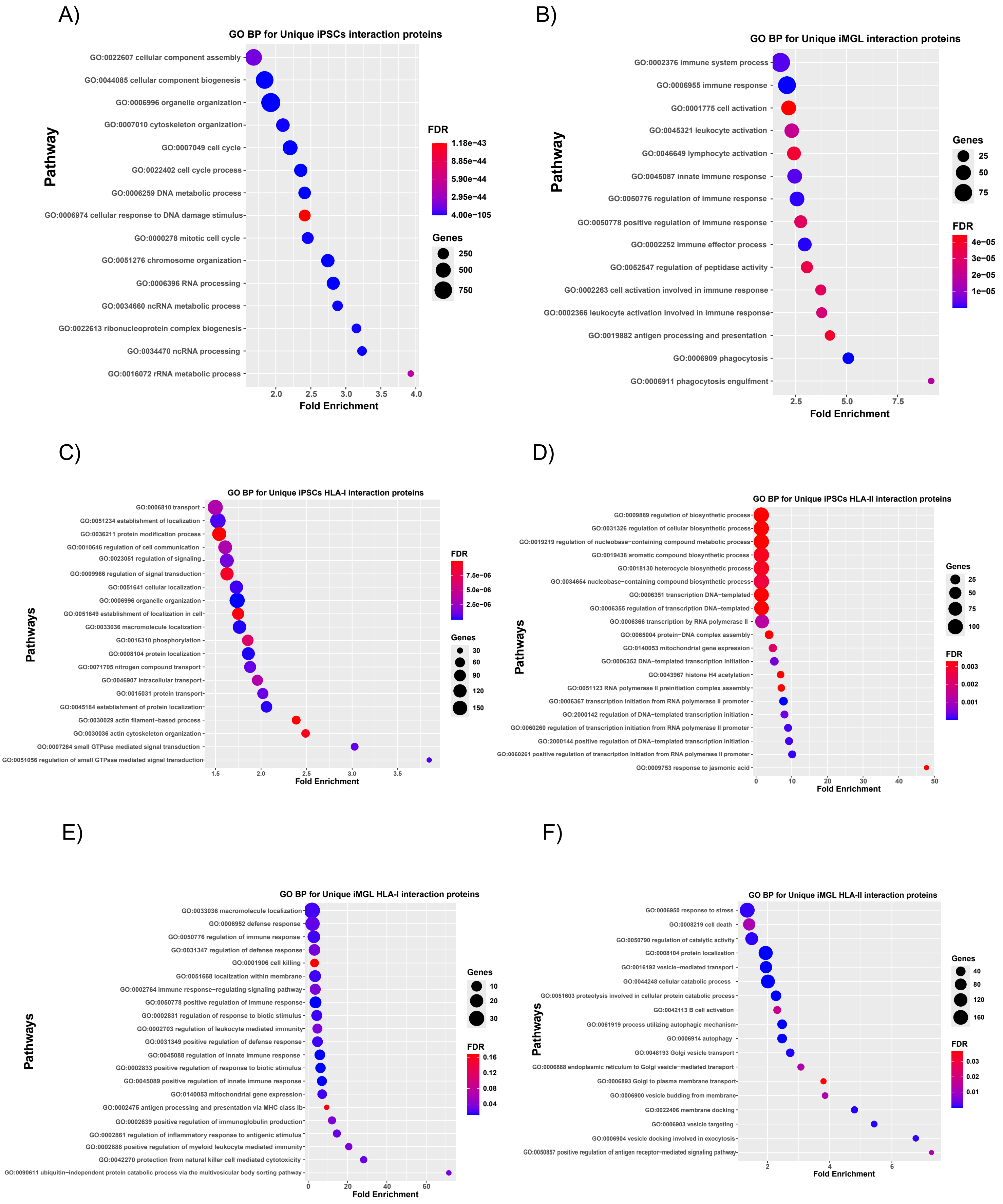

Supplementary Figure 5

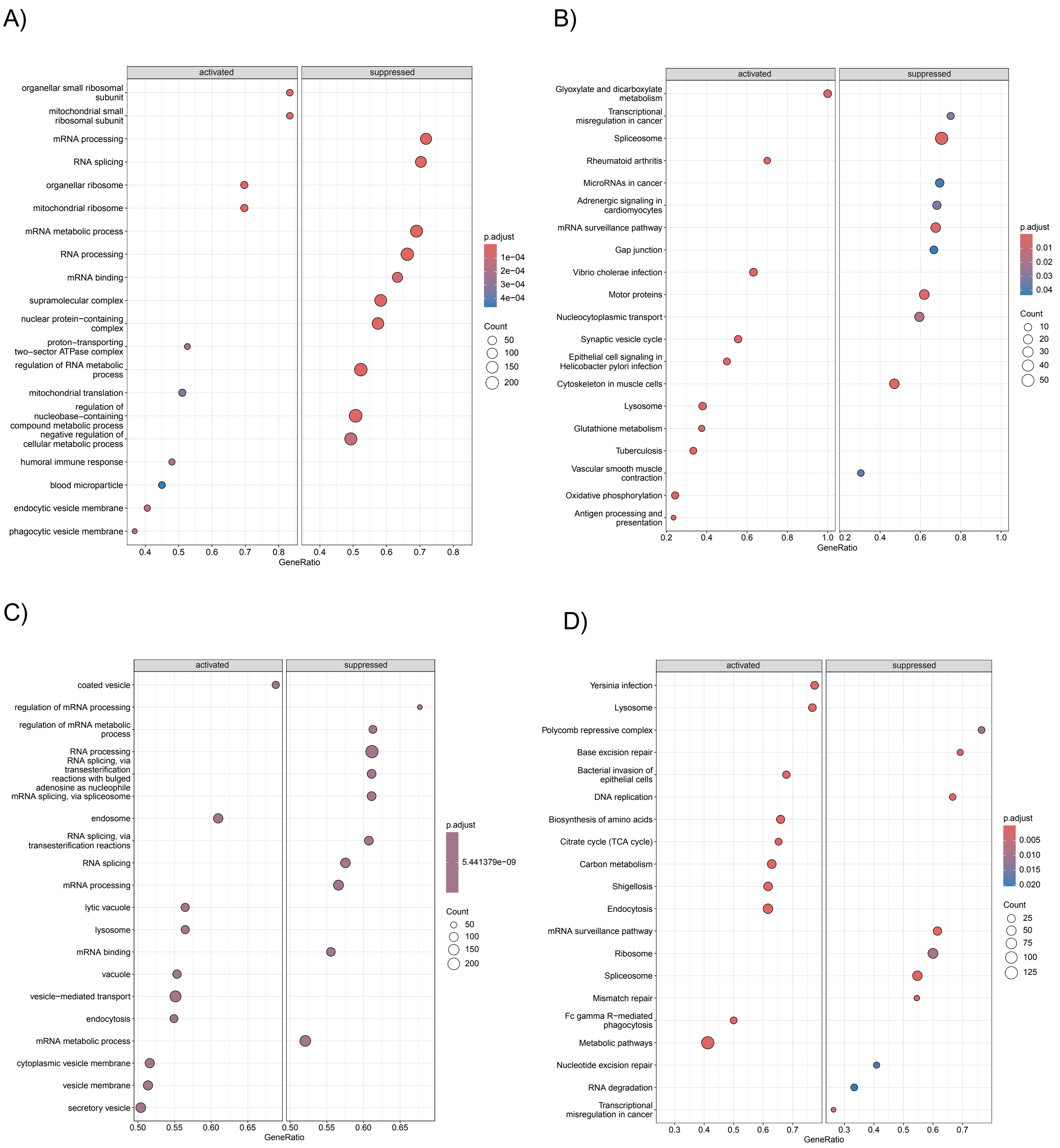

Supplementary Figure 6

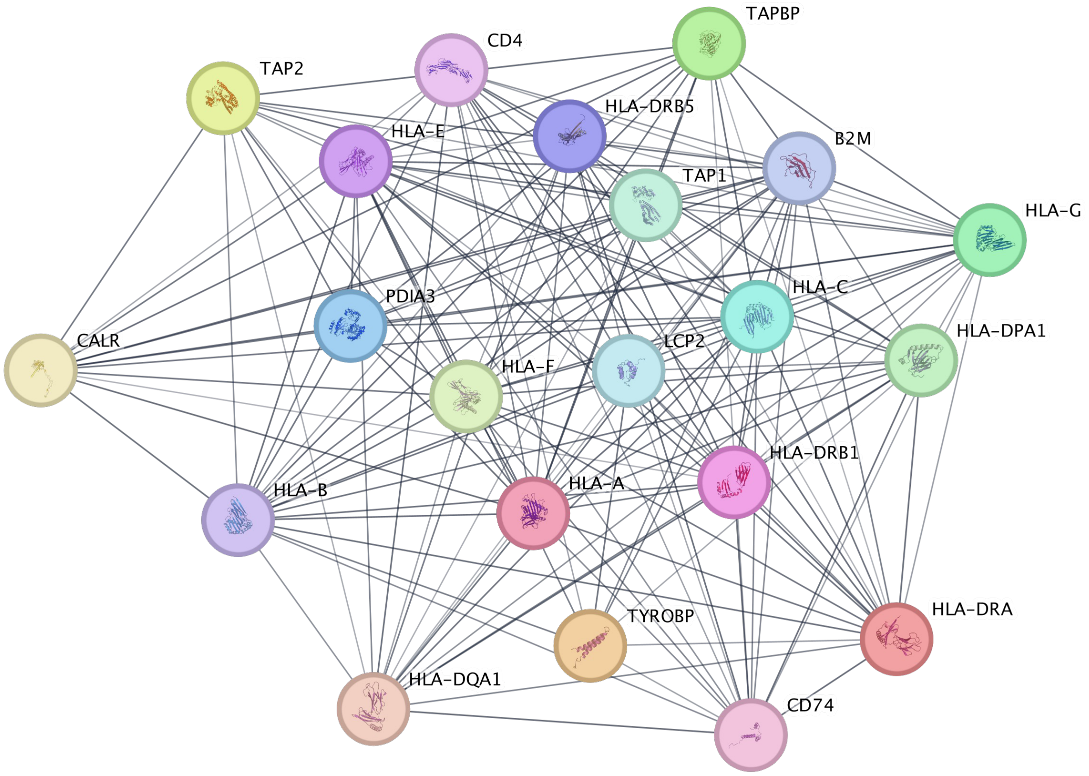

Supplement: Supplement 2 [file media-2.pdf]
